# Global Community Child Health

**DOI:** 10.3390/ijerph17093331

**Published:** 2020-05-11

**Authors:** Matthew Ellis, Puspa Raj Pant

**Affiliations:** 1 Centre for Academic Child Health, University of Bristol, 1-5 Whiteladies Road, Bristol BS8 1NU, UK; 2 Department of Nursing and Midwifery, University of the West of England (UWE Bristol), Bristol BS16 1QY, UK; puspa.pant@uwe.ac.uk

**Keywords:** Global Child Health, Community Child Health, SDGs, Social Determinants of Health

## Abstract

This special issue of IJERPH has published a range of studies in this developing field of Global Community Child Health research. A number of manuscripts submitted in response to our invitation describing ‘community-based interventions which impact on child health and wellbeing around the globe. In addition to rural community-based initiatives given that most children now live in cities we are also interested to hear about urban initiatives….’ We hope this issue will of great interest to the researchers and practitioners as well as academia from the fields of Global Health as well as Global Child Health because it comprised of 14 articles representing all five continents. Physical activity appears a key component of the scientific community’s current conception of child well-being judging from the four papers published addressing this area. This issue also has papers on childhood obesity to rubella vaccination. Despite of the journal’s strive for reaching out to a wider global child health community, this issue missed contributions relating to child safeguarding and social determinants of urban health.

Global community child health focusses on the health and development of children in a community context across the globe. Whilst some threats to the well-being of children may be globally determined (e.g., climate change) many others are more local (e.g., a busy trunk road). Whatever the level of the threat it is the mobilisation of community and household level interventions to protect and enable children which lies at the heart of global community child health [1]. Community health workers facilitate these interventions working with parents and carers [2] whilst schools, children’s centres, nurseries and creches provide enabling environments for interventions to reach children directly. Although we know that investment in early child development remains a top priority for all communities [3], it is becoming clearer that exclusive attention to the early years misses important opportunities both in middle childhood and the adolescent period [4].

This edition of IJERPH was conceived of as an opportunity to sample a range of studies in this developing field of research. We invited studies describing community-based interventions which impact on child health and wellbeing around the globe. In addition to rural community-based initiatives, given that most children now live in cities we are also interested to hear about urban initiatives. Although sustainable development goal (SDG) three was our primary focus, we were keen to hear about multi-sectoral interventions with synergistic impact across the SDGs.

The 14 accepted articles are global in their reach, with papers from all five inhabited continents. Physical activity appears to be a key component of the scientific community’s current conception of child well-being judging from the four papers published addressing this area [5,6,7,8]. Of course, given the global obesity epidemic, this will remain an important issue for community child health, though given the obesogenic environment we all face following societal nutrition transition we suspect the answers to this lie further upstream in the food industry’s regulatory framework [9].

Infectious disease, despite the epidemiological transition, remains a major threat in childhood everywhere and several aspects come up in this Special Issue—not least the awareness of a disease (rubella) amongst health care workers in Tanzania for which there is an available vaccine [10]. This reminds us that for vaccination, its understanding and promotion are key tasks for community health workers around the globe, even more so in this age of vaccine hesitancy. If there may be one benevolent side effect of Covid-19 going forward it may be the greater appreciation of the value of vaccines!

We also publish a paper presenting evidence in support of a role for a bacterial lysate to stimulate immunity in childhood [11] and an interesting exploration of traditional healers’ knowledge of Noma [12], the disfiguring facial erosion encountered in children in Africa, which almost certainly relates to the continuing wide spectrum of infectious disease in childhood. This paper reminds us that community health workers take many forms and a functional health system finds ways of connecting all members of the health care community.

Community mobilisation through groups is an important vehicle for community child health initiatives and where some of the best evidence of impact lies [13]. In this edition Fathers’ roles in parent groups supporting families affected by Zika virus [14] links well with our review of early intervention for infants at high risk of developmental disability [15]. A team working in Fiji also make use of group-based interventions in their description of what a community child health initiative looks like in an island community [16].

The social determinants of health are central to the concept of community child health [17]. These determinants operate at household, local population (“community”), national and supranational levels. We were sorry not to see any contributions relating to child safeguarding—always a sensitive and difficult research area—but one which therefore needs to be illuminated by an especially powerful light! This would be especially timely as we move globally towards legislation outlawing the corporal punishment of children (https://endcorporalpunishment.org/countdown).

Of course, the physical environment in which children play, go to school and all too often work also has a major impact on their health. Given that environmental health is a primary concern of this journal it was good to be able to accept two papers focussing on children, the first investigating the role of toys in the transmission of diarrhoeal disease at children’s centres in South Africa [18] and the second an exploratory study assessing pesticide levels in children’s urine in Mexico [19].

Strikingly, we did not receive a community-based study from an urban slum where far too many of the world’s children are growing up. If we are to promote "Health For All" at all ages then we must ensure that, as ”A Future for the World’s Children” [20] puts it, ”children grow up in safe and healthy environments, with clean water and air and safe spaces to play”. Research assessing the impact of community led initiatives into road traffic injury reduction, child safeguarding and the social determinants of health in urban slums should be a focus of community child health researchers going forward.

## References

[1] Rosato M., Laverack G., Grabman L.H., Tripathy P., Nair N., Mwansambo C., Azad K., Morrison J., Bhutta Z., Perry H. (2008). Community participation: Lessons for maternal, newborn, and child health. Lancet.

[2] WHO (2017). Integrated Management of Childhood Illness Global Survey Report.

[3] (2011). Editorial. Early child development-a winning combination. Lancet.

[4] Bundy D.A.P., de Silva N., Horton S., Patton G.C., Schultz L., Jamison D.T., Disease Control Priorities C., Adolescent H., Development Authors G. (2018). Investment in child and adolescent health and development: Key messages from Disease Control Priorities, 3rd Edition. Lancet.

[5] Dias K.I., White J., Jago R., Cardon G., Davey R., Janz K.F., Pate R.R., Puder J.J., Reilly J.J., Kipping R. (2019). International Comparison of the Levels and Potential Correlates of Objectively Measured Sedentary Time and Physical Activity among Three-to-Four-Year-Old Children. Int. J. Environ. Res. Public Health.

[6] Kowaluk A., Woźniewski M., Malicka I. (2019). Physical Activity and Quality of Life of Healthy Children and Patients with Hematological Cancers. Int. J. Environ. Res. Public Health.

[7] Müller I., Schindler C., Adams L., Endes K., Gall S., Gerber M., Htun N.S.N., Nqweniso S., Joubert N., Probst-Hensch N. (2019). Effect of a Multidimensional Physical Activity Intervention on Body Mass Index, Skinfolds and Fitness in South African Children: Results from a Cluster-Randomised Controlled Trial. Int. J. Environ. Res. Public Health.

[8] Rao Q., Bai L., LV Y., Abdullah A.S., Brooks I., Xie Y., Zhao Y., Hou X. (2020). Goal-Framing and Temporal-Framing: Effects on the Acceptance of Childhood Simple Obesity Prevention Messages among Preschool Children’s Caregivers in China. Int. J. Environ. Res. Public Health.

[9] Sonntag D., Schneider S., Mdege N., Ali S., Schmidt B. (2015). Beyond Food Promotion: A Systematic Review on the Influence of the Food Industry on Obesity-Related Dietary Behaviour among Children. Nutrients.

[10] Chotta N.A.S., Mgongo M., Uriyo J.G., Msuya S.E., Stray-Pedersen B., Stray-Pedersen A. (2019). Awareness and Factors Associated with Health Care Worker’s Knowledge on Rubella Infection: A Study after the Introduction of Rubella Vaccine in Tanzania. Int. J. Environ. Res. Public Health.

[11] Esposito S., Bianchini S., Polinori I., Principi N. (2019). Impact of OM-85 Given during Two Consecutive Years to Children with a History of Recurrent Respiratory Tract Infections: A Retrospective Study. Int. J. Environ. Res. Public Health.

[12] Baratti-Mayer D., Baba Daou M., Gayet-Ageron A., Jeannot E., Pittet-Cuénod B. (2019). Sociodemographic Characteristics of Traditional Healers and Their Knowledge of Noma: A Descriptive Survey in Three Regions of Mali. Int. J. Environ. Res. Public Health.

[13] Prost A., Colbourn T., Seward N., Azad K., Coomarasamy A., Copas A., Houweling T.A., Fottrell E., Kuddus A., Lewycka S. (2013). Women’s groups practising participatory learning and action to improve maternal and newborn health in low-resource settings: A systematic review and meta-analysis. Lancet.

[14] Smythe T., Duttine A., Vieira A.C.D., Castro B.d.S.M.d., Kuper H. (2019). Engagement of Fathers in Parent Group Interventions for Children with Congenital Zika Syndrome: A Qualitative Study. Int. J. Environ. Res. Public Health.

[15] Kohli-Lynch M., Tann C.J., Ellis M.E. (2019). Early Intervention for Children at High Risk of Developmental Disability in Low- and Middle-Income Countries: A Narrative Review. Int. J. Environ. Res. Public Health.

[16] Woolfenden S., Milner K., Tora K., Naulumatua K., Mataika R., Smith F., Lingam R., Kado J., Tuibeqa I. (2020). Strengthening Health Systems to Support Children with Neurodevelopmental Disabilities in Fiji—A Commentary. Int. J. Environ. Res. Public Health.

[17] Marmot M., Commission on Social Determinants of H. (2007). Achieving health equity: From root causes to fair outcomes. Lancet.

[18] Ledwaba S.E., Becker P., Traore-Hoffman A., Potgieter N. (2019). Bacterial Contamination of Children’s Toys in Rural Day Care Centres and Households in South Africa. Int. J. Environ. Res. Public Health.

[19] Sierra-Diaz E., Celis-de la Rosa A.d.J., Lozano-Kasten F., Trasande L., Peregrina-Lucano A.A., Sandoval-Pinto E., Gonzalez-Chavez H. (2019). Urinary Pesticide Levels in Children and Adolescents Residing in Two Agricultural Communities in Mexico. Int. J. Environ. Res. Public Health.

[20] Clark H., Coll-Seck A.M., Banerjee A., Peterson S., Dalglish S.L., Ameratunga S., Balabanova D., Bhan M.K., Bhutta Z.A., Borrazzo J. (2020). A future for the world’s children? A WHO-UNICEF-Lancet Commission. Lancet.

